# The Fibrotic Effects of TMAO on Human Renal Fibroblasts Is Mediated by NLRP3, Caspase-1 and the PERK/Akt/mTOR Pathway

**DOI:** 10.3390/ijms222111864

**Published:** 2021-11-01

**Authors:** Stefania Kapetanaki, Ashok Kumar Kumawat, Katarina Persson, Isak Demirel

**Affiliations:** 1 School of Medical Sciences, Campus USÖ, Örebro University, 701 82 Örebro, Sweden; ashok.kumawat@oru.se (A.K.K.); katarina.persson@oru.se (K.P.); isak.demirel@oru.se (I.D.); 2 Nephrology Department, Karolinska University Hospital, 171 76 Solna, Sweden; 3 Nephrology Department, Karolinska University Hospital, 141 86 Huddinge, Sweden; 4 Cardiovascular Research Center, School of Medical Sciences, Örebro University, 701 82 Örebro, Sweden; 5 iRiSC—Inflammatory Response and Infection Susceptibility Center, Faculty of Medicine and Health, Örebro University, 701 82 Örebro, Sweden

**Keywords:** TMAO, renal fibroblasts, proliferation, collagen, chronic kidney disease

## Abstract

Trimethylamine N-oxide (TMAO), a product of gut microbiota metabolism, has previously been shown to be implicated in chronic kidney disease. A high TMAO-containing diet has been found to cause tubulointerstitial renal fibrosis in mice. However, today there are no data linking specific molecular pathways with the effect of TMAO on human renal fibrosis. The aim of this study was to investigate the fibrotic effects of TMAO on renal fibroblasts and to elucidate the molecular pathways involved. We found that TMAO promoted renal fibroblast activation and fibroblast proliferation via the PERK/Akt/mTOR pathway, NLRP3, and caspase-1 signaling. We also found that TMAO increased the total collagen production from renal fibroblasts via the PERK/Akt/mTOR pathway. However, TMAO did not induce fibronectin or TGF-β1 release from renal fibroblasts. We have unraveled that the PERK/Akt/mTOR pathway, NLRP3, and caspase-1 mediates TMAO’s fibrotic effect on human renal fibroblasts. Our results can pave the way for future research to further clarify the molecular mechanism behind TMAO’s effects and to identify novel therapeutic targets in the context of chronic kidney disease.

## 1. Introduction

Trimethylamine N-oxide (TMAO) is the result of the oxidation of trimethylamine (TMA) primarily by the enzyme flavin-containing monooxygenase 3 in the liver. It can also be generated by the oxidation of TMA that takes place in the gut microbiota [1]. TMA is produced by gut residing microbes using diet compounds such as choline, betaine, L-carnitine, ergothioneine, and gamma-butyrobetaine as precursors. These precursors are mainly obtained from dairy products, fish, shrimp, red meat, wheat, and beans. Urine is the main excretion route of both TMAO and TMA out from the body [1,2].

Physiologically, TMAO has a variety of properties in order to facilitate the homeostasis of organisms. It physically interacts with proteins and acts as a stabilizer of their folded state. Moreover, it is a natural osmolyte that neutralizes cellular perturbations resulting from changes in osmolarity, urea, and hydrostatic pressure. The most representative example is the urine-concentrating cells of the kidney medulla. TMAO protects these cells from cell death caused by intracellularly accumulating urea [3,4]. Finally, TMAO is an electron acceptor during the anaerobic metabolism of Enterobacteriaceae, which are bacteria of the human gut flora [4].

TMAO has been implicated in a spectrum of diseases, but the strongest association is to cardiovascular and kidney disease. Increased TMAO has prognostic value for all-cause mortality in patients with peripheral arterial disease [5,6]. In addition, high levels of TMAO are associated with increased cardiovascular risk [7]. TMAO also has a role in the progression of atherosclerosis, as there is positive correlation between plasma levels of TMAO and the size of the atherosclerotic plaque in the aorta [8,9]. In addition, TMAO contributes to the development of atherosclerosis by promoting macrophage transformation into foam cells [8,9]. Patients with chronic kidney disease (CKD) and high TMAO have an increased risk of cardiovascular events and increased mortality [10,11]. TMAO is independently associated with kidney function [12,13]. Patients with end-stage renal disease have increased levels of TMAO and TMA [14], which probably depends on their decreased plasma clearance as a consequence of a low glomerular filtration rate (GFR). However, TMAO may also be released from the renal medulla secondary to kidney ischemic injury [15,16]. Elevated plasma levels of TMAO are associated with a poor prognosis in CKD patients [17], higher incidence of hospitalizations in hemodialysis patients [18], and reduced survival [13]. A CKD mouse model fed iodomethylcholine, an indirect TMAO inhibitor that suppresses TMA generation, exhibited reduced levels of renal injury markers such as urea, fibroblast growth factor 23 (FGF23), and cystatin C [19]. The latter marker of functional impairment was also increased in TMAO-fed mice. The elevated TMAO in those mice was associated with tubulointerstitial fibrosis and collagen deposition in histopathologic kidney samples [17]. These results suggest a causal relationship between TMAO and CKD development and progression [17,19].

Renal fibrosis leads to nephron loss and progressively declined renal function. Detection of myofibroblasts in histopathologic kidney samples is a prognostic index for fibrosis progression and progression of tubular atrophy [20]. Both lead to end-stage kidney disease (ESKD). Sunsaku et al. identified a variety of molecular biomarkers which correlate with tubulointerstitial fibrosis that can be used as therapeutic targets and predictors of progressive renal disease [21]. The NLRP3 inflammasome has been associated with the development of fibrosis in several diseases, including kidney disease [22,23]. In kidney disease, the NLRP3 inflammasome has been shown to contribute to the progression of acute kidney injury, chronic kidney disease, and diabetic nephropathy [24,25,26,27]. Today, there is no data implying a direct connection of a specific molecular pathway with the effect of TMAO on the human renal interstitium. The aim of this study was firstly to identify the fibrotic effects of TMAO on human renal fibroblasts and secondly, to unravel which molecular pathways mediate these effects.

## 2. Results

### 2.1. TMAO Induces Renal Fibroblast Activation

**Figure 1:**
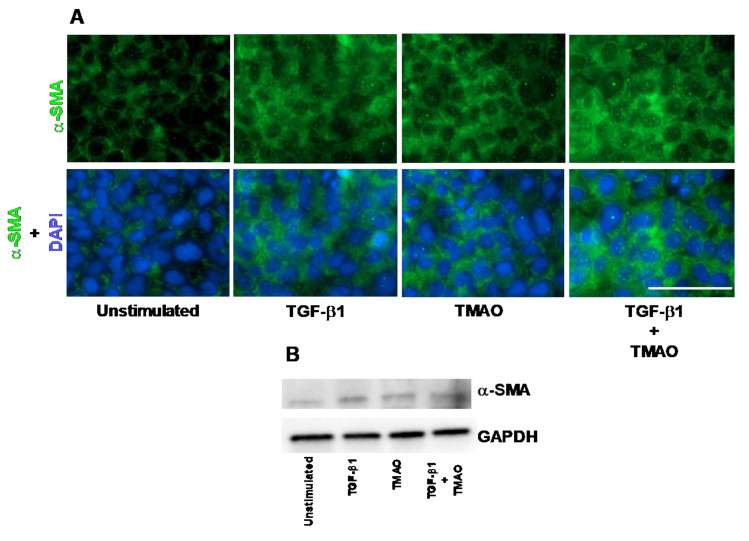
Trimethylamine N-oxide (TMAO) promotes renal fibroblast activation. Renal fibroblasts were stimulated with 300 µM TMAO and 10 ng/mL TGF-β1 for 24 h and α-SMA expression was visualized with fluorescence microscopy (**A**) and Western blot (**B**). Green represents α-SMA (smooth muscle actin) and blue (DAPI) represents the nucleus. Scale bar represents 100 µm. GAPDH was used as a loading control. Data are representative of three independent experiments.

The human renal fibroblast cell line TK173 was stimulated with TMAO and TGF-β1 and the expression of α-SMA was assessed. We found that the expression of α-SMA was increased in renal fibroblasts after treatment with TMAO for 24 h compared to unstimulated cells, as detected with immunofluorescence and Western blot (Figure 1A,B). We also found that TGF-β1 increased the protein expression of α-SMA compared to unstimulated cells (Figure 1A,B). Combination treatment with both TMAO and TGF-β1 had no synergistic effect (Figure 1A,B). Taken together, these findings show that TMAO is able to activate renal fibroblasts upon stimulation.

### 2.2. TMAO Promotes Renal Fibroblast Proliferation

**Figure 2:**
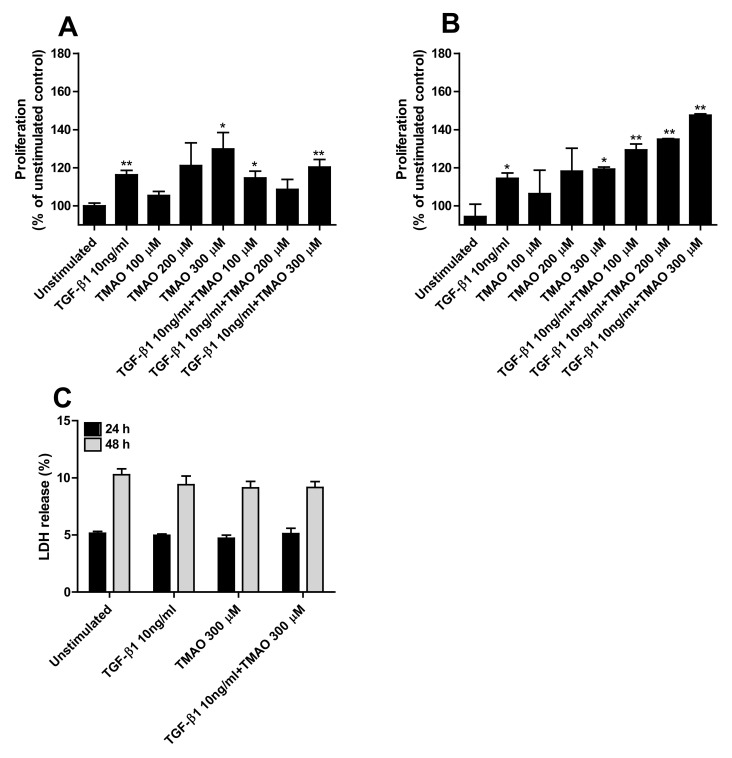
TMAO induces renal fibroblast proliferation. Renal fibroblasts were stimulated with 100–300 µM TMAO and 10 ng/mL TGF-β1 for 24 h (**A**,**C**) or 48 h (**B**,**C**) and proliferation (**A**,**B**) or LDH release (**C**) were evaluated. Proliferation is presented as % of unstimulated control. LDH release is presented as % of total LDH. Data are presented as mean ± SEM (*n* = 3 independent experiments). Asterisks denote statistical significance compared to unstimulated cells (* *p* < 0.05, ** *p* < 0.01).

We continued to investigate if TMAO could increase the proliferation of renal fibroblasts. We found that TMAO induced a dose-dependent increase in proliferation of renal fibroblasts after 24 h (Figure 2A) and 48 h (Figure 2B) of exposure. However, only 300 µM TMAO induced a significant increased proliferation compared to unstimulated cells (Figure 2A,B). Stimulation with the fibrotic agent TGF-β1 also induced a significantly increased proliferation compared to unstimulated cells (Figure 1A,B). The combination of both TMAO and TGF-β1 had a proliferative effect on renal fibroblasts, although not synergistic (Figure 2A,B). Moreover, no significant differences were noted regarding cell death (LDH release) between unstimulated and TMAO stimulated cells (Figure 2C).

### 2.3. TMAO Increases the Proliferation of Renal Fibroblasts via the PERK/Akt/mTOR Pathway

**Figure 3:**
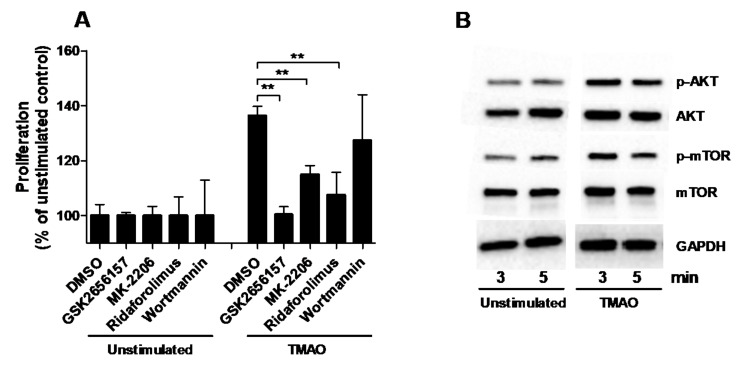
PERK/Akt/mTOR pathway mediates TMAOs proliferative effect on renal fibroblasts. Renal fibroblasts were pre-incubated with DMSO (vehicle), PERK inhibitor GSK2656157 (0.5 µM), Akt inhibitor MK-2206 (1 µM), mTOR inhibitor ridaforolimus (1 µM) or PI3K inhibitor wortmannin (1 µM) for 1 h prior to TMAO stimulation (300 µM) for 48 h (**A**) followed by evaluating proliferation. Proliferation is presented as % of unstimulated control. Western blot analysis was conducted to identify differences in protein levels of p-Akt/Akt and p-mTOR/mTOR after TMAO (300 µM) stimulation for 3 and 5 min (**B**). GAPDH was used as a loading control. Data are presented as mean ± SEM (*n* = 3 independent experiments). Asterisks denote statistical significance (** *p* < 0.01).

The next step in our investigation was to identify the signaling pathway through which TMAO exerts its effect on the proliferation of renal fibroblasts. We found that inhibition of Akt (MK-2206) and mTOR (Ridaforolimus), but not PI3K (Wortmannin), resulted in significantly reduced fibroblast proliferation compared to DMSO treated cells after TMAO stimulation for 48 h (Figure 3A). We also found that inhibiting PERK (GSK2656157) reduced the TMAO-induced proliferation of renal fibroblasts (Figure 3A). In addition, Western blot results showed that renal fibroblasts expressed higher levels of p-Akt and p-mTOR after 3 min and 5 min of TMAO exposure compared to unstimulated fibroblasts (Figure 3B). However, we did not see any higher levels of p-Akt and p-mTOR after 15 min or 30 min (data not shown).

### 2.4. TMAO-Induced Proliferation of Renal Fibroblasts Is Mediated by NLRP3 and Caspase-1

**Figure 4:**
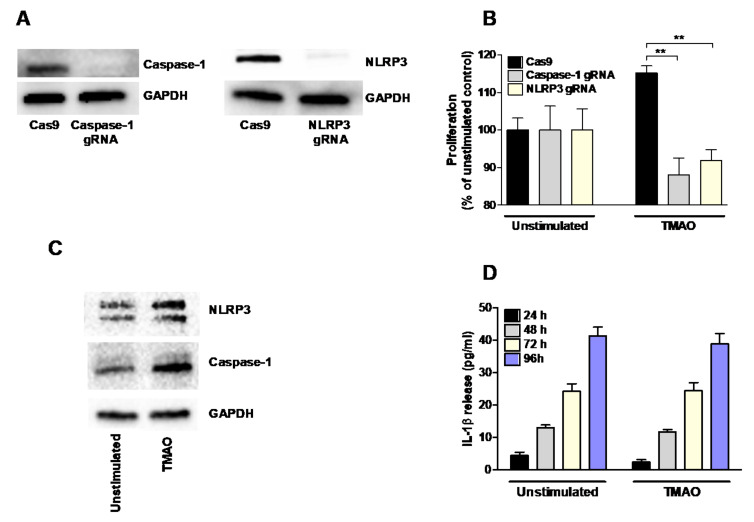
NLRP3 and caspase-1 mediate TMAO’s proliferative effect on renal fibroblasts. NLRP3 and caspase-1 KO CRISPR Cas9 renal fibroblasts were constructed and evaluated by Western blot (**A**). The NLRP3 and caspase-1 KO cells were stimulated with 300 µM TMAO and the proliferation was assessed after 48 h (**B**). Proliferation is presented as % of unstimulated control. Western blot analysis was conducted to evaluate NLRP3 and caspase-1 levels after TMAO (300 µM) stimulation for 48 h (**C**). IL-1β release was quantified from renal fibroblasts following 24–96 h stimulation with TMAO (300 µM) (**D**). GAPDH was used as a loading control. gRNA stands for guideRNA targeting specific genes using CRISPR/Cas9. Data are presented as mean ± SEM (*n* = 3 independent experiments). Asterisks denote statistical significance (** *p* < 0.01).

Initially, NLRP3 and caspase-1 knockout (KO) renal fibroblast cell lines were constructed using the CRISPR/Cas9 system. The absence of NLRP3 and caspase-1 was verified using Western blot (Figure 4A). The NLRP3 and caspase-1 KO cells were stimulated with TMAO for 48 h and the proliferation was investigated. TMAO induced a significantly lower proliferation of the NLRP3, and caspase-1 KO cells compared to the TMAO stimulated Cas9 control cells (Figure 4B). Western blot analysis showed that TMAO-stimulated renal fibroblasts expressed higher levels of NLRP3 and caspase-1 compared to the unstimulated cells after 48 h (Figure 4C). However, no increased release of IL-1β was observed after TMAO stimulation from renal fibroblasts compared to unstimulated cells after 24–96 h (Figure 4D). These results suggest that NLRP3 and caspase-1 are involved in the proliferative effect of TMAO on renal fibroblasts.

### 2.5. TMAO Has no Effect on the Production of Fibronectin or TGF-β1 from Renal Fibroblasts

**Figure 5:**
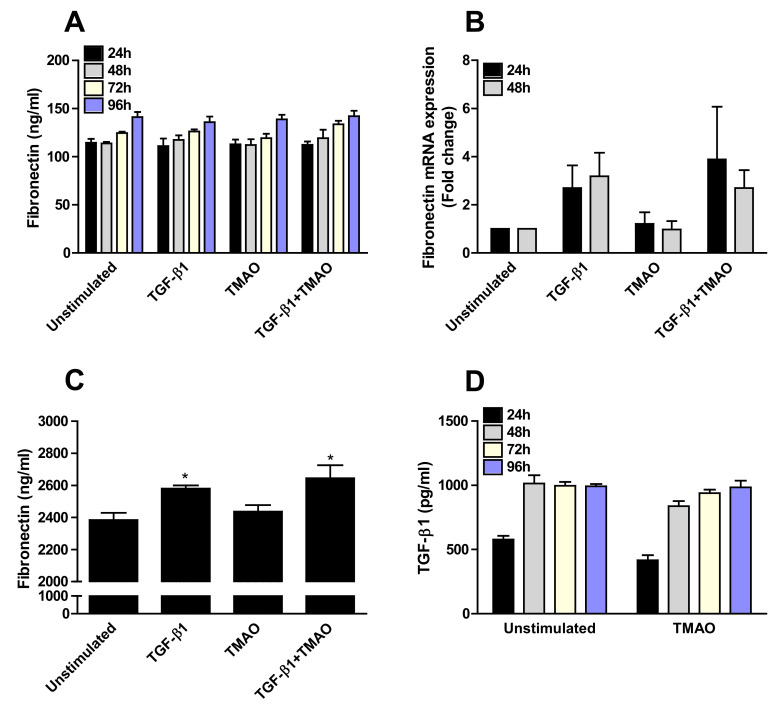
TMAO stimulation does not induce fibronectin or TGF-β1 production from renal fibroblasts. Renal fibroblasts were stimulated with 300 µM TMAO and 10 ng/mL TGF-β1 for 24 h (**A**,**B**,**D**), 48 h (**A**,**B**,**D**), 72 h (**A**,**D**) or 96 h (**A**,**C**,**D**) and fibronectin release (**A**,**C**) fibronectin gene expression (**B**) and TGF-β1 release (**D**) were evaluated. Fibronectin levels in supernatants in combination with cell lysates were also evaluated (**C**). Data are presented as mean ± SEM (*n* = 3 independent experiments). Asterisks denote statistical significance compared to unstimulated cells (* *p* < 0.05).

TMAO stimulation of renal fibroblasts caused no increased fibronectin secretion (Figure 5A) or mRNA expression (Figure 5B) compared to unstimulated cells. Similar results were found after TGF-β1 stimulation at the protein level (Figure 5A). However, a small but not significant increased gene expression of fibronectin was observed after TGF-β1 stimulation (Figure 5B). Next, we investigated the presence of fibronectin in the cell lysates and supernatants together, as fibronectin is known to be anchored to integrins on the cell membrane [28]. We found that TGF-β1 stimulation, but not TMAO, significantly increased the protein expression of fibronectin compared to unstimulated cells (Figure 5C). Furthermore, we also found that TMAO stimulation did not induce an increased TGF-β1 release from renal fibroblasts compared to unstimulated cells (Figure 5D), indicating that TMAO exerts its effects on renal fibroblasts directly and not using the fibrotic agent TGF-β1 as a mediator.

### 2.6. TMAO Increases Total Collagen Production via the Akt/mTOR Pathway

**Figure 6:**
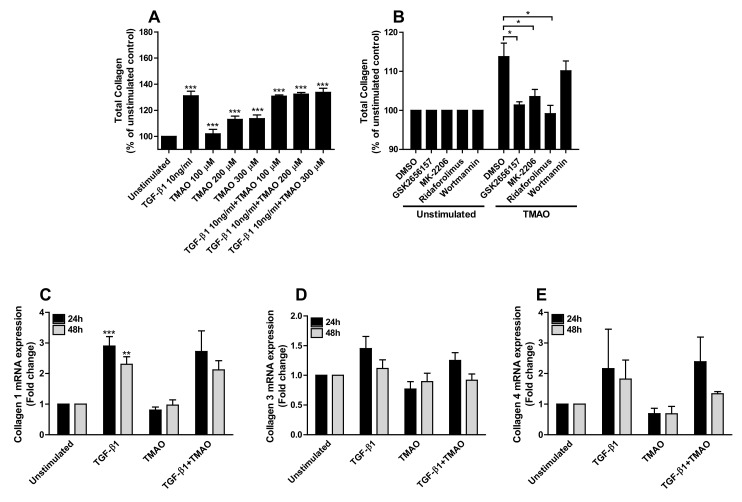
TMAO increases total collagen production from renal fibroblasts through the PERK/Akt/mTOR pathway. Renal fibroblasts were stimulated with 100–300 µM TMAO and 10 ng/mL TGF-β1 for 96 h and total collagen production was evaluated (**A**). Renal fibroblasts were also pre-incubated with DMSO (vehicle), PERK inhibitor GSK2656157 (0.5 µM), Akt inhibitor MK-2206 (1 µM), mTOR inhibitor ridaforolimus (1 µM) or PI3K inhibitor wortmannin (1 µM) for 1 h prior to TMAO stimulation (300 µM) for 96 h (**B**) followed by evaluating total collagen production. Total collagen is presented as % of unstimulated control. Real-time RT-PCR was conducted to detect mRNA expression of collagen 1 (**C**), 3 (**D**), and 4 (**E**) following TMAO (300 µM) and TGF-β1 10 ng/mL stimulation for 24–48 h. Data are presented as mean ± SEM (*n* = 3 independent experiments). Asterisks denote statistical significance compared to unstimulated cells (* *p* < 0.05, ** *p* < 0.01, *** *p* < 0.001).

Increased total collagen expression from renal fibroblasts was observed after 96 h of TMAO stimulation in a dose-dependent manner compared to unstimulated cells (Figure 6A). Increased total collagen expression was also found upon TGF-β1 stimulation. No additive or synergistic effect was exhibited upon stimulation with TGF-β1 in combination with TMAO (Figure 6A). We found that inhibition of Akt (MK-2206) and mTOR (ridaforolimus), but not PI3K (wortmannin), resulted in significantly reduced total collagen expression compared to DMSO treated cells after TMAO stimulation for 96 h (Figure 6B). We also found that inhibiting PERK (GSK2656157) reduced the TMAO-induced collagen expression (Figure 6B). However, we found that TMAO did not increase the gene expression of collagen types 1, 3, or 4 after 24–48 h compared to unstimulated cells. Only TGF-β1 induced significantly increased expression of collagen type 1 compared to unstimulated cells (Figure 6C–E).

## 3. Discussion

Several studies have investigated the role of TMAO in fibrosis development in various diseases [17,19,29,30,31]. In the kidneys, renal fibrosis leads to nephron loss and progressively declined renal function. Detection of myofibroblasts in histopathologic kidney samples is a prognostic index for fibrosis progression and progression of tubular atrophy [20]. Both lead to end-stage kidney disease (ESKD). However, today there are no data linking specific molecular pathways with the effect of TMAO on human renal fibrosis. Our aim was, therefore, to investigate the fibrotic effects of TMAO on renal fibroblast and to elucidate the molecular pathways involved.

We started by evaluating if TMAO could activate human renal fibroblasts into myofibroblasts. Myofibroblasts are characterized by increased α-SMA expression, high proliferation rate, and increased production of extracellular matrix (ECM) components such as collagen and fibronectin [32,33,34]. We found that TMAO induced renal fibroblast activation as indicated by the increased α-SMA level in TMAO-treated renal fibroblasts. This activation was at least as strong as the TGF-β1-mediated increase of α-SMA. It is known that resident fibroblasts of the renal interstitium get differentiated to myofibroblasts as a response to growth factors such as TGF-β1, FGF, IL-1, PDGF, TNF-α, and aldosterone [20]. TGF-β1 promotes the activation of myofibroblasts, their persistence in the site of injury, and the expression of ECM, namely fibronectin and collagen [33,34,35,36]. Our findings indicate that TMAO is a strong renal fibroblast activator.

Next, we proceeded with evaluating the effect of TMAO on renal fibroblast proliferation, collagen, fibronectin, and TGF-β1 production. We found that TMAO increased fibroblast proliferation equivalent to TGF-β1-mediated proliferation. We also found that TMAO increased total collagen production from renal fibroblasts, but not fibronectin or TGF-β1 production. This indicates that TMAO does not mediate its fibrotic effect through TGF-β1 release. To our knowledge, there are no studies that have elucidated the fibrotic mechanism of TMAO at the molecular level in human renal fibroblasts. Our findings show that the Akt/mTOR pathway mediates the signaling by which TMAO exerts its collagen-producing and proliferative effect on renal fibroblasts. Our findings show that TMAO increased the phosphorylation of Akt and mTOR but did not affect their total protein level. At the functional level, the Akt (MK-2206) and mTOR (ridaforolimus) inhibitors significantly inhibited TMAO-induced proliferation and collagen production. However, the PI3K inhibitor (wortmannin) did not reduce TMAO-induced proliferation. Looking at the gene expression of collagens, TMAO did not induce an increased gene expression of collagen 1, 3, or 4, which have previously been associated with renal fibrosis [37,38]. This suggests that the increase of total collagen may be an effect of the increased proliferation of renal fibroblasts induced by TMAO. The PI3K/Akt/mTOR pathway has a variety of biological effects on cells both at the physiological and pathological levels. At the physiological level, it promotes cell viability, prevents apoptosis, and induces autophagy in erythropoiesis [39,40]. In addition, it is involved in cell proliferation and cell fate determination [41,42,43,44,45]. At the pathological level, its role is established in neurodegenerative disease, tumor growth, tumor cells proliferation, and metabolism [39,46]. There is a variety of recent studies on biological agents targeting PI3K, Akt, and mTOR to treat hematological malignancies and solid tumors [47,48,49,50,51,52,53,54,55]. Much research exists on the newly identified plant derivatives that use the PI3K/Akt/mTOR pathway as a mediator to affect fibroblast apoptosis [56,57] or proliferation [58]. Taken together, our findings indicate that only Akt and mTOR, but not PI3K, mediates the effect of TMAO on collagen production and human renal fibroblast proliferation.

Recently TMAO was found to directly bind to and activate protein kinase R-like endoplasmic reticulum kinase (PERK), an ER stress kinase in hepatocytes. The study suggested that PERK was a TMAO receptor [59]. In our findings, we observed that inhibition of PERK reduced the TMAO-mediated collagen production and proliferation of renal fibroblasts. It has been shown, in agreement with our findings, that activated PERK can mediate the activation of the PI3K/Akt/mTOR pathway through its lipid kinase activity. PERKs lipid kinase activity converts diacylglycerol to phosphatidic acid (PA), and PA is important for mTOR complex formation and Akt activation [60,61,62,63]. This shows that there is a link between PERK and mTOR/Akt in collagen production and renal fibroblast proliferation.

We also investigated whether NLRP3 inflammasome activation could be involved in TMAO-induced fibroblast proliferation. A variety of studies support the association of the NLRP3 inflammasome with fibrosis, TMAO, Akt and mTOR [22,23,64,65,66,67]. Using NLRP3 and caspase-1 knockout cell lines, we found that the proliferative effect of TMAO on human renal fibroblasts is NLRP3 and caspase-1 dependent. We also found increased protein levels of NLRP3 and caspase-1 after TMAO treatment. However, TMAO stimulation of renal fibroblasts did not induce the release of IL-1β, indicating that the role of NLRP3 and capsase-1 in TMAO-mediated fibroblast proliferation is independent of NLRP3 inflammasome activation. It has previously been shown that NLRP3 via an inflammasome-independent role mediates renal injury and contributes to the progression of chronic kidney disease [68,69]. According to Artlett et al., chronically activated NLRP3 inflammasome leads to constant ECM synthesis and induction of fibrosis by maintaining fibroblasts in their activated state or by keeping a high level of TGF-β1 via IL-1β production [22,23]. Moreover, there is evidence that NLRP3 inflammasome has a role in lung [23] and liver [64] fibrosis. Regarding the effect of TMAO on NLRP3 inflammasome, it has been shown that the TMAO increases the activation of the NLRP3 inflammasome in carotid arteries of mice [65]. Studies have also shown that PERK, mTOR, and Akt are involved in NLRP3 inflammasome activation. PERK was shown to induce NLRP3 inflammasome activation via NF-κB, and PERK silencing was found to decrease the protein expression of NLRP3 in hepatocytes [70,71,72]. Furthermore, Akt and mTOR are known to regulate NLRP3 inflammasome activation [66,67]. Taken together, our findings show that NLRP3, caspase-1 and the PERK/Akt/mTOR pathway are all involved in TMAO-mediated renal fibroblast proliferation.

**Figure 7:**
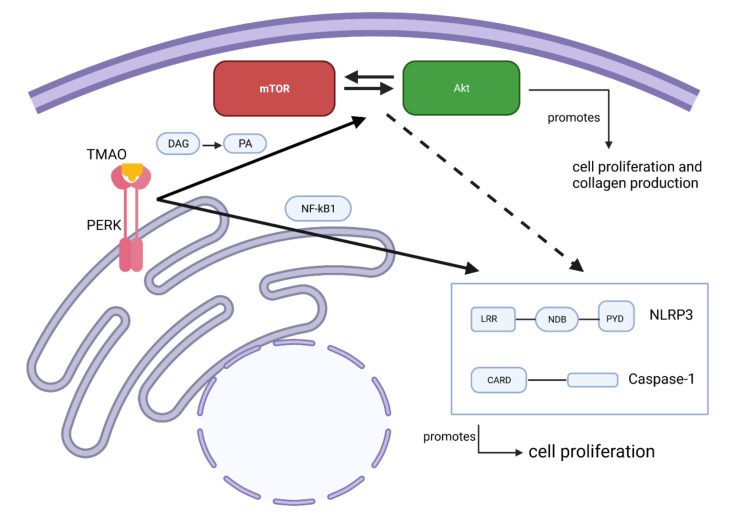
Proposed molecular mechanism of the effect of TMAO on renal fibroblasts. PERK is an intracellular TMAO receptor. This could be true in renal fibroblasts, as shown in the current study. Phosphorylated PERK is proposed to trigger the mTOR complex formation via diacylglycerol (DAG) conversion to phosphatidic acid (PA). mTOR can either activate or be activated by Akt. The Akt/mTOR pathway then promotes cell proliferation and collagen production. Alternatively, the same pathway can, as previously shown, regulate NLRP3 and caspase-1 and, indirectly, affect cell proliferation. Activated PERK can directly affect NLRP3 via NF-κB activation.

In conclusion, our findings showed that TMAO promotes renal fibroblast activation, proliferation, and collagen production via PERK/Akt/mTOR pathway, NLRP3, and caspase-1 signaling. To the best of our knowledge, this is the first study unraveling that the PERK/Akt/mTOR pathway, NLRP3, and caspase-1 mediate TMAO’s fibrotic effect on human renal fibroblasts (summarized in Figure 7). Our results can be the basis of further research to elucidate the molecular mechanism behind TMAOs effect and to identify novel therapeutic targets in the context of CKD.

## 4. Materials and Methods

### 4.1. Cell Culture

Human renal medullary fibroblast cell line TK173 (a kind gift from Professor Anton Jan van Zonneveld, Leiden University, Leiden, The Netherlands) was used [73]. The TK173 cell line was cultured in Dulbecco’s modified eagle medium (DMEM, Lonza, Basel, Switzerland) supplemented with 10% fetal bovine serum (FBS), 2 mM L-glutamine, and 1 mM non-essential amino acids (all from Thermo Fisher Scientific, Waltham, MA, USA) at 37 °C in a humidified incubator with 5% CO_2_. The cells were serum-starved overnight in DMEM supplemented with 2 mM L-glutamine and 1 mM non-essential amino acids prior to experiments. During the experiments, the medium was replaced by DMEM supplemented with 2 mM L-glutamine, 1 mM non-essential amino acids, and 1% FBS or 0% FBS, depending on the experimental setup.

### 4.2. CRISPR/Cas9 Genome Editing of Renal Fibroblasts

CRISPR/Cas9 gene editing in the TK173 cells was conducted using the pSpCas9 (BB)-2A-Puro (PX459, V2.0) (a gift from Feng Zhang, Addgene plasmid #62988) [74] plasmid. Plasmid transfection was done using Lipofectamine 2000 (Life Technologies, Carslbad, CA, USA). The target sites were: GCTAATGATCGACTTCAATG (NLRP3) and GACAGTATTCCTAGAAGAAC (caspase-1). The TK173 cells were selected with puromycin (2.5 µg/mL; Sigma-Aldrich, St. Louis, MO, USA) 24 h after transfection. All experiments were done with a polyclonal pool of gene-edited cells. The gene editing was confirmed at the protein level by Western blot analysis.

### 4.3. Stimulation of Renal Fibroblasts

Renal fibroblasts were stimulated with TMAO (100 µM, 200 µM, or 300 µM; Sigma-Aldrich) for 24–96 h, depending on the experimental setup, at 37 °C in 5% CO_2_. As a positive control, renal fibroblasts were stimulated with TGF-β1 (10 ng/mL Invivogen, CA, USA). The renal fibroblasts were also pre-incubated with DMSO (vehicle), PERK inhibitor GSK2656157 (0.5 µM, Santa Cruz Biotechnology Inc., Heidelberg, Germany), Akt inhibitor MK-2206 (1 µM, Selleckchem, Houston, TX, USA), mTOR inhibitor ridaforolimus (1 µM, Selleckchem), and PI3K inhibitor wortmannin (1 µM, Selleckchem) for 1 h prior to TMAO stimulation. Supernatants were collected and kept at −80 °C until further analysis.

### 4.4. Immunofluorescence

The renal fibroblasts were stimulated with 300 µM TMAO and 10 ng/mL TGF-β1 for 24 h and incubated at 37 °C with 5% CO_2_. After stimulation, the fibroblasts were washed with PBS and fixed for 15 min in 4% paraformaldehyde. Fibroblasts were then permeabilized using 0.1% Triton X-100 in PBS for 10 min. The cells were then blocked to prevent unspecific antibody binding by using 1% bovine serum albumin (BSA) for 30 min. Human α-SMA was detected by using a mouse monoclonal anti-α-SMA antibody (Santa Cruz Biotechnology) diluted 1:100, for 1 h (in PBS with 1% BSA). A secondary goat polyclonal anti-mouse A488 conjugated antibody (Abcam, Cambridge, UK), diluted 1:1000, was used for 1 h (in PBS with 1% BSA). The nucleus was stained using 4′,6-diamidino-2-phenylindole (DAPI; Santa Cruz Biotechnology) for 10 min. Samples were evaluated with the Cytation 3 plate reader microscope (BioTek, Winooski, VT, USA), as previously described [24].

### 4.5. Crystal Violet Proliferation Assay

The renal fibroblasts were stimulated with 300 µM TMAO and 10 ng/mL TGF-β1 for 24 h and 48 h and incubated at 37 °C with 5% CO_2_. After stimulation, the fibroblasts were washed once with PBS and 0.1% Crystal violet (Sigma-Aldrich) diluted in 20% methanol was added to the cells. The cells were incubated for 10 min at room temperature and then washed twice with tap water. The cells were then destained with 1% sodium dodecyl sulfate (SDS) on a shaker at 500 rpm for 5 min. The optic density (OD) was measured using the Cytation 3 plate reader at 570 nm.

### 4.6. Western Blot Analysis

The renal fibroblasts were lysed by scraping the cells in radioimmunoprecipitation assay (RIPA) buffer supplemented with phosphatase inhibitor cocktail (Thermo Fisher Scientific). The protein concentration in each sample was measured using the DC protein assay (Bio-Rad Laboratories, Hercules, CA, USA). Equal amounts of sample and Laemmli buffer were mixed and boiled for 5 min in 95 °C. The samples (10 µg of protein) were separated with 4–15% SDS-polyacrylamine gel electrophoresis and then transferred to a polyvinylidene fluoride (PVDF) membrane (Bio-Rad Laboratories). The PVDF membrane was blocked with 3% BSA for 1 h at room temperature. Phospho-Akt (p-Akt) was detected using a rabbit monoclonal antibody (Cell Signaling Technologies, Danvers, MA, USA). Phospho-mTOR (p-mTOR) was detected using a rabbit monoclonal antibody (Cell Signaling Technologies). Total Akt was detected using a mouse monoclonal antibody (Cell Signaling Technologies). Total mTOR was detected using a rabbit monoclonal antibody (Cell Signaling Technologies). NLRP3 was detected using a rabbit monoclonal antibody (Cell Signaling Technologies). Caspase-1 was detected using a mouse monoclonal antibody (AdipoGen Life Sciences, Buckingham, UK). GAPDH was used as a loading control and was detected with a rabbit polyclonal antibody (Santa Cruz Biotechnology). All the primary antibodies were incubated overnight at 4 °C. The secondary antibodies, goat anti-mouse IgG (horseradish peroxidase, HRP) (Abcam) and goat anti-rabbit IgG (HRP) (Abcam) were used and incubated for 1 h at room temperature. Luminata Forte Western HRP Substrate (Merck Millipore, Burlington, MA, USA) was used for developing the blots, as previously described [24].

### 4.7. Measurement of IL-1β, Fibronectin, TGF-β1 Release and Cell Viability

IL-1β, fibronectin, and TGF-β1 release from renal fibroblasts was analyzed by enzyme-linked immunosorbent assay (ELISA). IL-1β was quantified using the human IL-1β kit (ELISA MAX™ Deluxe Sets, BioLegend, San Diego, CA, USA). Fibronectin was quantified using the human fibronectin kit (Duo set, ELISA, R&D Systems, Minneapolis, MN, USA). TGF-β1 was quantified using the human TGF-β1 kit (R&D Systems). Cell viability was assessed by Pierce lactate dehydrogenase (LDH) cytotoxicity assay (Thermo Fisher Scientific) following the manufacturer’s protocol [75]. The OD for all assays was evaluated using the Cytation 3 plate reader.

### 4.8. Quantification of Total Collagen Production

The renal fibroblasts were stimulated with 300 µM TMAO and 10 ng/mL TGF-β1 in the presence of 50 µg/mL sodium ascorbic acid (Thermo Fisher Scientific) for 96 h incubated at 37 °C with 5% CO_2_. After stimulation, total collagen production was assessed using Sirius red staining (Thermo Fisher Scientific). The supernatants from the culture wells were removed and 1 mg/mL Sirius red (diluted in picric acid) was added to the cells and incubated for 30 min at room temperature. The cells were then washed with PBS and destained with NaOH 0.1 M on a shaker at 700 rpm for 15 min at room temperature. The destaining solutions were then transferred to a new 96-well plate and the OD was measured at 540 nm with the Cytation 3 plate reader.

### 4.9. RNA Isolation and Real-Time RT-PCR

Total RNA was isolated from human renal fibroblasts using the E.Z.N.A. Total RNA Kit I (Omega Bio-tek, Norcross, GA, USA) following the manufacturer’s instructions. Determination of the RNA yield was done using spectrophotometry (Nano-Drop ND-1000, Wilmington, NC, USA). First strand cDNA synthesis was performed using 100 ng total RNA with the High capacity cDNA RT kit (Thermo Fisher Scientific). The real-time RT-PCR was conducted using Maxima SYBR Green qPCR Master Mix (Thermo Fisher Scientific), 10 ng cDNA and 250 nM of each primer (Table 1). The primers were designed by Origene (Rockville, MD, USA) and synthesized by Eurofins MWG Synthesis GmbH (Munich, Germany). The amplification of the PCR was done using CFX96 Touch Real-Time PCR Detection System (Bio-Rad Laboratories). The protocol used was as follows: desaturation at 95 °C for 10 min, 40 cycles of denaturation at 95 °C for 15 s, and finally, annealing/extension at 60 °C for 60 s. The mRNA expression was assessed by the comparative Ct (ΔΔCt) method followed by normalization to the endogenous control GAPDH. Fold difference was calculated as 2^−^^ΔΔCt^, as previously described [24].

### 4.10. Data Analysis

All data shown are expressed as mean ± SEM. The differences between the groups were analyzed by one-way ANOVA followed by Bonferroni multiple testing correction. Statistical significance of the differences was considered at *p* < 0.05.

## Figures and Tables

**Table 1:** Primers used in the real-time qPCR.

Gene	Oligonucleotide Sequences (5′–3′)
*Fibronectin*	F: ACAACACCGAGGTGACTGAGAC R: GGACACAACGATGCTTCCTGAG
Collagen 1	F: GATTCCCTGGACCTAAAGGTGC R: AGCCTCTCCATCTTTGCCAGCA
Collagen 3	F: TGGTCTGCAAGGAATGCCTGGA R: TCTTTCCCTGGGACACCATCAG
Collagen 4	F: TGTTGACGGCTTACCTGGAGAC R: GGTAGACCAACTCCAGGCTCTC
GAPDH	F: GTCTCCTCTGACTTCAACAGCG R: ACCACCCTGTTGCTGTAGCCAA

## Data Availability

Not applicable.
